# Protocol for the Creation of the Guidelines International Network–McMaster Guideline Development Checklist Extension for Integrating Artificial Intelligence in the Guideline Enterprise (Guidelines‐Artificial Intelligence Extension)

**DOI:** 10.1002/gin2.70038

**Published:** 2025-08-22

**Authors:** Manuel Marques‐Cruz, Bernardo Sousa‐Pinto, Wojtek Wiercioch, Marge Reinap, Ignacio Neumann, Yuan Chi, Artur Nowak, Mariette Awad, Monika Nothacker, Jan Brozek, Pablo Alonso‐Coello, Amir Qaseem, Elie A. Akl, Holger J. Schünemann

**Affiliations:** ^1^ MEDCIDS ‐ Department of Community Medicine, Information and Health Decision Sciences, Faculty of Medicine University of Porto Porto Portugal; ^2^ MEDCIDS CINTESIS@RISE ‐ Health Research Network, Faculty of Medicine University of Porto Porto Portugal; ^3^ Michael G. DeGroote Cochrane Canada & McMaster GRADE Centres McMaster University Hamilton Ontario Canada; ^4^ Department of Health Research Methods, Evidence and Impact McMaster University Hamilton Ontario Canada; ^5^ WHO Regional Office for Europe Copenhagen Denmark; ^6^ School of Medicine Universidad San Sebastián Santiago Chile; ^7^ Evidence Prime Hamilton Ontario Canada; ^8^ Department of Electrical and Computer Engineering American University of Beirut Beirut Lebanon; ^9^ AWMF (Association of the Scientific Medical Societies in Germany) Institute for Medical Knowledge Management University of Marburg Marburg Germany; ^10^ Institut de Recerca Sant Pau (IR SANT PAU) Barcelona Spain; ^11^ Centro de Investigación Biomédica en Red de Epidemiología y Salud Pública (CIBERESP) Madrid Spain; ^12^ American College of Physicians Philadelphia Pennsylvania USA; ^13^ Department of Internal Medicine American University of Beirut Beirut Lebanon; ^14^ Clinical Epidemiology and Research Center (CERC), IRCCS Humanitas Research Hospital Humanitas University Milan Italy; ^15^ Fraunhofer Institute for Translational Medicine and Pharmacology ITMP, Allergology and Immunology Berlin Germany

**Keywords:** artificial intelligence, guidelines, protocol

## Abstract

**Introduction:**

Artificial intelligence (AI) has the potential to support processes of the guideline enterprise. To support the adequate preplanning and the transparent reporting of the use of AI in the guideline enterprise, the Guidelines International Network (GIN) proposed the development of an extension of the GIN–McMaster Guideline Development Checklist (GDC) for the use of AI in guidelines. Here we describe the protocol for the development of this extension.

**Methods:**

We will follow a multiphase approach to generate an extension of the GIN–McMaster GDC. We will start by prompting a large language model to suggest relevant items for the extension. We will subsequently revise the obtained outputs in an iterative process involving the different elements of the working group. This iterative process will be informed by a scoping review. We will then reach different stakeholders to test the proposed checklist extension on their easiness of use, implementation, feasibility and reasonability.

**Questions:**

This protocol lays the ground for the development of an extension to the GIN–McMaster GDC for the use of AI in guidelines. This extension will support the adequate use of AI in the guideline enterprise.

## Summary

- This article describes a protocol for the creation of an extension for the GIN–McMaster Guideline Development Checklist for the use of artificial intelligence in guidelines.
- The development of the checklist extension will encompass several phases, including a scoping review, interaction of artificial intelligence for preliminary item generation, iterative refinement of the items, internal consensus, user testing and final approval.
- The checklist extension for the use of artificial intelligence in guidelines will support the adequate use of artificial intelligence in the guideline enterprise.

## 1 Introduction

There is virtually no area in medicine and health care delivery that the use of artificial intelligence (AI) is not already being explored. In the guideline enterprise, AI has the potential to support the processes of planning, development and adaptation, reporting, implementation, impact evaluation, certification and appraisal of recommendations. In fact, several studies have been published using AI‐based tools or processes to support specific tasks of the guideline enterprise, such as question generation or evidence gathering for generation of recommendations [1, 2].

The increasing importance that AI may display in the guideline enterprise has led the Guidelines International Network (GIN) board to propose a set of principles for the use of AI tools or processes to support the health guideline enterprise [3]. One of those principles concerned transparency and reported that ‘To ensure transparency in the use of AI in the guideline enterprise, all applied AI tools, data sources and methodologies should be clearly documented and made—as much as possible—accessible and understandable to all users’ [3]. To support the implementation of that principle, the GIN board proposed the development of an extension of the GIN–McMaster Guideline Development Checklist (GDC) for the use of AI in guidelines [3].

The GIN–McMaster GDC for guideline developers has become the standard for considering all aspects in guideline development [4]. It is endorsed by GIN, a charity established in 2002, which connects more than 100 guideline developing member organisations. Following the publication of the GIN–McMaster GDC, several extensions have been published or are under development, including for rapid guidelines [5], considerations of equity [6], adaptation [7] and for the integration of quality improvement and quality assurance [8]. Here we describe the protocol for developing an extension of the GIN–McMaster GDC for integrating AI in the guideline enterprise. Target users of this extension include those involved in the planning, development, implementation, dissemination and updating of guidelines.

## 2 Methods

GIN has approved the creation of a working group on the role of AI in the context of guidelines to address the underlying opportunities, risks and limitations (https://g-i-n.net/get-involved/working-groups). The working group started with 14 guideline developers (including three members of the GIN board) from most regions of the world with backgrounds in evidence‐based medicine and guideline development and/or data science and AI. The current paper will be an official working group document.

### 2.1 Study Design

**Figure 1:**
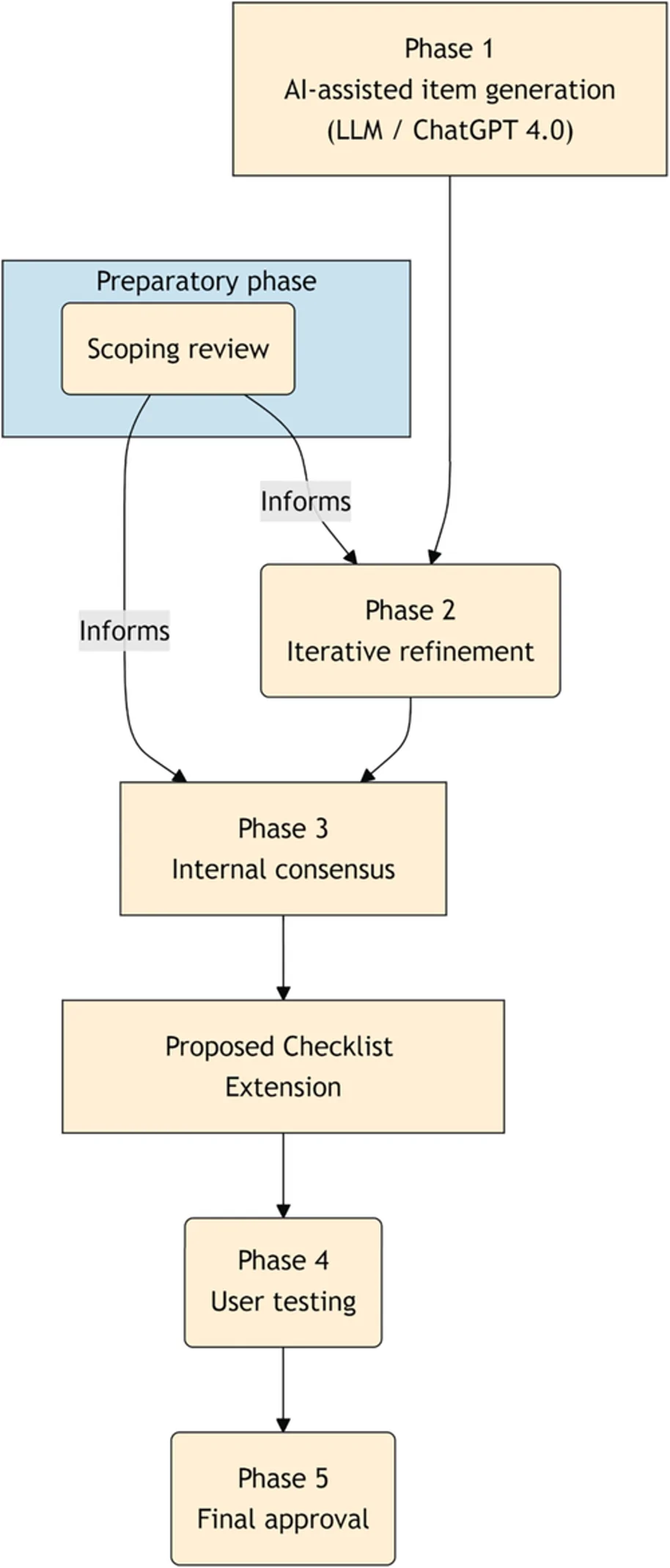
Development phases of GIN checklist extension for integrating AI in the guideline cycle. AI = artificial intelligence, LLM = large language models.

We will follow a multiphase approach to generate a list of items concerning the integration of AI tools in the guideline enterprise based on the original GDC and its 18 steps. First, we will prompt a large language model (LLM, specifically ChatGPT 4.0) to suggest relevant items on the use of AI tools for each guideline development step (phase 1). ChatGPT 4.0 was chosen given that, in a previous study conducted by some elements of our team in which several LLMs were assessed, it was the one displaying the best performance (Marques‐Cruz et al. Use of artificial intelligence to support the assessment of the methodological quality of systematic reviews. 2025. Under Review). Outputs obtained from the LLM will be extensively revised in two moments: (i) an iterative refinement (phase 2) in which two elements of the working group will independently assess and delete, modify or add items based on the output received; and (ii) internal evaluation (phase 3) through consensus by all working group members, that will assess the items obtained from the iterative refinement, in combination with the results of the scoping review, and collectively assess the inclusion of those items or of additional items. Phases 2 and 3 will be informed by a scoping review (preparatory phase) that will be conducted in parallel by the members of the GIN working group on the use of AI tools in the guideline enterprise. The resulting proposal for checklist extension will be tested for easiness of use, implementation, feasibility and reasonability by end users (phase 4), through public consultation. The final version of the checklist extension will then be assessed by the working group for final approval (phase 5), and, dependant on approval of that final version, published in a scientific journal. The full study design is shown in Figure 1.

### 2.2 Preparatory Phase: Scoping Review

We will conduct a scoping review of the current use of AI tools in the guideline enterprise [3]. We will clarify the current definition of AI tools used in the guideline enterprise. We will follow The University of Adelaide Joanna Briggs Institute (JBI) Scoping Review Network Resources, including the JBI Manual for Evidence Synthesis [9] and adhere to the Preferred Reported Items for Systematic reviews and Meta‐Analyses Extension for Scoping Reviews for reporting [10].

In the scoping review, we will address (i) the definitions, principles and rationale for AI use in the guideline enterprise, (ii) the current uses for AI tools in the guideline enterprise, and (iii) the barriers and facilitators of AI tools used. Therefore, the scoping review will (i) inform on the existence of eventual other checklists for the use of AI in the guideline enterprise, and (ii) capacitate members of the GIN working group to more adequately revise the output obtained from the LLM.

The full protocol of the scoping review has been published elsewhere [3]. In brief, scoping review will be based on a search of Medline and EMBASE, as well as on an assessment of grey literature by forward citation search. Its eligibility criteria will include (i) relevant systematic reviews on AI tool use in any step of the guideline enterprise; (ii) primary studies on AI uses in the guideline enterprise with detailed steps, including those using AI tools as a major step for the guideline development or implementation; (iii) guidelines that acknowledge the use of AI tools in their development; and (iv) methodological research on the integration of AI tools in the guideline enterprise. The selection process will involve the independent assessment of each record by two researchers (first, by title and abstract screening and second, by full text reading), with disagreements being solved by consensus. As per the methodology of scoping reviews, risk of bias assessment will not be performed. We will perform a qualitative summary of the results—for each topic that the scoping review aims to address, we will create a table with the unique aspects mentioned in the included primary studies, the respective references and the guideline websites (e.g., a table with the barriers and facilitators of AI tools identified in primary studies, alongside with the references which mentioned those barriers and facilitators).

### 2.3 Phase 1: AI‐Assisted Item Generation

In this phase, we will prompt ChatGPT 4.0 for a list of suggested relevant items related to the integration of AI tools in each of the 18 guideline development domains of the original GDC [4]. Items from the GDC and items from the GIN–McMaster Checklist Extension for Quality Assurance and Quality Improvement [8] will be provided as context. The prompt structure to be used is described in Box 1. Data on the total number of suggested items per guideline development step will be provided.

Box 1 **Prompt structure for obtaining guideline extension items with ChatGPT 4.0.**

**System:** You are a helpful assistant. Your role is to propose all relevant topic‐specific guideline development items to a list of pre‐specified steps. A user will provide a topic and a guideline development step. You will answer with the items, separated by ‘;’ when more than one, or with ‘None’ if no additional items are relevant.

**User:** Topic—General/Nonspecific; Guideline development step—[*one of the 18 development steps*]

**Assistant:** [*all items in the GDC for the specified development step*]

**User:** Topic—Integrating Quality Assurance and Improvement in guideline development; Guideline development step—[*one of the 18 development steps*]

**Assistant:** [*all items in the GDC for the specified development step*]

**User:** Topic—Integrating Artificial Intelligence tools in guideline development; Guideline development step—[*one of the 18 development steps*]

### 2.4 Phase 2: Iterative Refinement

In this phase, two members of the working group will independently assess the suggested items. First, a comparison of the proposed items with the GDC and Quality Assurance and Quality Improvement extension checklist will be performed and duplicates will be removed. Suggested items that do not refer to the use of AI tools for the guideline enterprise will be deleted. The remaining items will be revised and further decisions on deletions or modifications will be made. Lastly, additional relevant items will be proposed by the two reviewers. The items resulting from this iterative refinement will be defined by consensus between the two investigators based on their previous independent work. In case consensus is not reached, a discussion with a third senior researcher will be performed. A description of the number of items deleted, modified and added will be provided.

This phase will be complemented by results of the scoping review, particularly those of other eventual checklists for the use of AI in guidelines (we will use an abstraction form to retrieve items from those checklists). In addition, this phase will be informed by the experience of the members of the working group in having participated in the scoping review.

### 2.5 Phase 3: Internal Consensus

In this step, an assessment of the items obtained from the iterative refinement will be performed by all working group members. A purposely built item feedback form will be provided to the members. Each member will be asked to decide on the inclusion or exclusion of each suggested item from phase 2, and will be able to propose modifications—with justifications grounded on both relevance and feasibility—to the existing items or additional items that they may find relevant. Since the main idea of this checklist extension is to be as complete as possible, this modification step should be held until no new ideas for items appear. A meeting will be held after this stage to achieve a checklist extension proposal by consensus. For items for which no consensus is reached, a formal voting process will be implemented, with need for at least 80% of participants providing the same vote for each item for a decision to be made. A description of the number of items deleted, modified and added will be provided. This phase will be informed by the experience of the members of the working group in having participated in the scoping review.

### 2.6 Phase 4: User Testing

Feedback on the proposed checklist extension will be sought through public consultation by end users. Prospective users of this guideline extension include the World Health Organization and the National Institute for Health and Care Excellence (NICE) and other Guideline Development Groups. We will survey members of such guideline panels about their opinion on whether the proposed items are adequate to describe AI integration in the guideline enterprise and to report the experience and suggestions on the proposed checklist extension (including regarding the time spent applying the checklist), which will be used to assess easiness of use, implementation, feasibility and reasonability. We will also invite other relevant stakeholders for public consultation through e‐mail and GIN social media and survey infrastructure as well as the GRADE Working Group LinkedIn and Teams channels, including authors of published uses of AI in the guideline enterprise. Results of the survey will be presented as aggregate data for closed‐ended and open‐ended questions using standard descriptive statistics and graphical representations (e.g., bar charts). For some open‐ended questions, anonymised representative answers will be presented. We plan to modify the checklist extension according to the provided feedback obtained using semi‐structured interviews. If there are any newly emerged items, we will ask for and deliberate carefully regarding further inclusion within core working group.

### 2.7 Phase 5: Final Approval

The working group members will compile the final version of the revised checklist extension and deliberate on its final approval. The final checklist extension for integrating AI in the guideline development enterprise will be included in a manuscript to be published in a scientific journal with previously published guideline checklists or checklist extensions.

## 3 Discussion

AI can bring important gains to the guideline enterprise. The GIN working group on AI has classified the potential gains resulting from the use of AI into three different groups: (A) performance of tasks that would have not been feasible by humans alone with the available resources, (B) provision of complementary inputs to those provided by humans, and (C) increase in the efficiency of tasks [3]. However, AI tools can also be inadequately used in the guideline context (e.g., use of models with poor performance or use of outputs generated by AI without adequate human critical appraisal, particularly considering that large language models can produce incorrect or misleading information, particularly when faced with ambiguous or incomplete data [11]), with potential negative consequences. Therefore, it is crucial that the use of AI in the guideline enterprise is reported in a transparent way, so that such use can be comprehensively appraised. This is all more important considering that human values and biases are able to directly or indirectly influence the output of AI‐based models [12].

This protocol lays the ground for the development of an extension of the GIN–McMaster GDC for the use of AI in guidelines according to the recently published GIN principles for such use [3]. In addition to the expected gains in transparency resulting from the use of this tool, we anticipate that the process leading to its development will itself contribute to relevant knowledge advances. In fact, the scoping review that will inform the development of the checklist extension will potentially identify research gaps (and, henceforth, areas for future research) in the use of AI tools in the guideline enterprise. On the other hand, the use LLMs as a complementary tool in item generation will provide a valuable case study on (i) how AI‐generated insights can be incorporated into the guideline cycle; and (ii) on the limitations of taking AI outputs at face.

AI is a quickly evolving field and its use in the guideline enterprise is relatively recent. We expect a wealth of emerging research demonstrating and discussing how AI tools can be used in different stages of the guideline enterprise. Therefore, the GDC extension we aim to develop is not to be interpreted as ‘a definitive document’, but rather as a guide which is to be subject to continuous evaluation and revision.

## Author Contributions

Manuel Marques‐Cruz and Bernardo Sousa‐Pinto participated in conceptualisation, methodology and writing – original draft; Wojtek Wiercioch, Marge Reinap, Ignacio Neumann, Yuan Chi, Artur Nowak, Mariette Awad, Monika Nothacker, Jan Brozek, Pablo Alonso‐Coello, Amir Qaseem and Elie A. Akl participated in methodology and writing – review and editing; Holger J. Schünemann participated in conceptualisation, supervision and writing – review and editing.

## Ethics Statement

The authors have nothing to report.

## Conflicts of Interest

Artur Nowak and Jan Brozek are employed and shareholders of Evidence Prime.

## Data Availability

This is a protocol and no data were generated or collected.
